# Datasets of genes coexpressed with FBN1 in mouse adipose tissue and during human adipogenesis

**DOI:** 10.1016/j.dib.2016.06.055

**Published:** 2016-07-05

**Authors:** Margaret R. Davis, Erik Arner, Cairnan R.E. Duffy, Paul A. De Sousa, Ingrid Dahlman, Peter Arner, Kim M. Summers

**Affiliations:** a The Roslin Institute, University of Edinburgh, Easter Bush, EH25 9RG, UK; b RIKEN Center for Life Science Technologies (Division of Genomic Technologies) (CLST (DGT)), 1-7-22 Suehiro-cho, Tsurumi-ku, Yokohama, Kanagawa 230-0045, Japan; c Centre for Clinical Brain Sciences, University of Edinburgh, Chancellors Building, 49 Little France Crescent, Edinburgh, EH16 4SB, UK; d Department of Medicine, Huddinge (Med H), Karolinska Universitetssjukhuset Huddinge, 141 86 Stockholm, Sweden

## Abstract

This article contains data related to the research article entitled “Expression of *FBN1* during adipogenesis: relevance to the lipodystrophy phenotype in Marfan syndrome and related conditions” [1]. The article concerns the expression of *FBN1*, the gene encoding the extracellular matrix protein fibrillin-1, during adipogenesis *in vitro* and in relation to adipose tissue *in vivo*. The encoded protein has recently been shown to produce a short glucogenic peptide hormone, (Romere et al., 2016) [2], and this gene is therefore a key gene for regulating blood glucose levels. *FBN1* and coexpressed genes were examined in mouse strains and in human cells undergoing adipogenesis. The data show the genes that were coexpressed with *FBN1*, including genes coding for other connective tissue proteins and the proteases that modify them and for the transcription factors that control their expression. Data analysed were derived from datasets available in the public domain and the analysis highlights the utility of such datasets for ongoing analysis and hence reduction in the use of experimental animals.

**Specifications Table**

**Table:** 

Subject area	*Biology*
More specific subject area	*Formation of adipose tissue from mesenchymal stem cells and adipocyte precursors*
Type of data	*Figures, Excel file*
How data was acquired	1. *A list of mouse genes that were correlated with Fbn1 was derived from BioGPS* (http://biogps.org, *accesssed 18 June 2015) using the “Correlation” tab, with a minimum correlation coefficient for inclusion of 0.70* 2. *Human data for a time course of adipogenesis in the FANTOM5 project* (http://fantom.gsc.riken.jp/zenbu/) *were downloaded on 8 May 2015 and clustered using BioLayout Express^3D^ Version 3.2 (available for download at* http://www.biolayout.org). *Clusters of coexpressed genes are presented in order of cluster size* 3. *Human data for expression of 1380 transcription factor genes during the adipogenesis time course were downloaded from the FANTOM5 project* (http://fantom.gsc.riken.jp/zenbu/) *on 28 May 2015 and clustered using BioLayout Express^3D^* (http://www.biolayout.org). *Clusters of coexpressed genes are presented in order of size*
Data format	*Analysed*
Experimental factors	*The mouse analysis used data from a previous study*[3]*in which RNA was extracted from epididymal adipose tissue from 28 inbred mouse strains. Mice were* 25 *weeks of age. RNA from two to four mice per strain was pooled*
	*The human analysis used data from the FANTOM5 project*[4]*. Human mesenchymal stem cells were cultured in the presence of adipogenic medium for up to 14 days*[5], [6], [7]*. Expression results for time points at induction of differentiation and 3 h, 1, 2, 4, 8, 12 and 14 days after induction of differentiation were downloaded for this analysis. Further details are available at*http://fantom.gsc.riken.jp/5/sstar/MSC_to_adipocyte_(human)*.*
Experimental features	1. *Mouse data. The record for the mouse Fbn1 gene was accessed at* http://biogps.org [8]. *The dataset chosen was eQTL – Fat (GNF1M). The probeset was gnf1m00711_a_at. Under the “Correlation” tab, a correlation cutoff of 0.75 was chosen. The list of genes was downloaded and is presented in the spreadsheet* 2. *Human data. Gene-based expression levels using promoter analysis by cap analysis of gene expression (CAGE)* [9] *were obtained from* http://fantom.gsc.riken.jp/zenbu/ *for the mesenchymal stem cell to adipocyte differentiation experiment. 7633 genes showing non-zero expression in at least one time point were analysed using BioLayout Express3D with a correlation coefficient cutoff of 0.85 and an MCL inflation value of 2.2* [10], [11] 3. *Human transcription factor data. Gene-based expression levels based on promoter analysis by CAGE were obtained from* http://fantom.gsc.riken.jp/zenbu/ *for the mesenchymal stem cell to adipocyte differentiation experiment. Expression patterns for 1380 genes were clustered with a correlation coefficient cutoff of 0.62 and an MCL inflation value of 2.2*
Data source location	*n/a*
Data accessibility	*The data are included with this article*

**Value of the data**

• Adipogenesis involves the downregulation of generic mesenchymal extracellular matrix (ECM) genes and upregulation of those genes encoding proteins specific to adipose tissue. The data provide a comprehensive list of the genes whose expression is altered during adipogenesis, which will assist in finding gene networks that are up- and downregulated during this process.

• The mouse data focus on fibrillin-1, the precursor of a newly described glucogenic hormone. In the mouse, *Fbn1* mRNA varies between strains and the data present a list of genes whose expression is correlated with that of *Fbn1 in vivo* (in mice). These data provide insight into the function of fibrillin-1 in adipogenesis and can be used also to determine the appropriate mouse strains for further experiments on obesity.

• The data also show how transcription factors are altered during the process of adipogenesis in human cells, which allows researchers to determine the regulatory molecules that are likely to control expression of key genes.

• These data will be useful in determining key genes downregulated during adipogenesis and can be compared with other experiments and *in vivo* data from humans and other animals.

## 1 Data

The main data consist of gene lists. The first list contains genes whose expression was correlated with that of *Fbn1* in different mouse strains. The second list shows all the clusters derived by BioLayout *Express*^3D^ for the differentiation of mesenchymal stem cells to adipocytes. Each cluster represents a different pattern of expression, which is described in the table. The patterns show a transition from generalised mesenchymal extracellular matrix to a specialised adipose extracellular matrix, with high expression of mitochondrial and lipid processing genes by the end of the time course. The third list shows the clusters of transcription factors derived from the same experiment. A similar transition in transcription factor expression was observed as adipogenesis progressed.

## 2 Experimental design, materials and methods

**Fig. 1:**
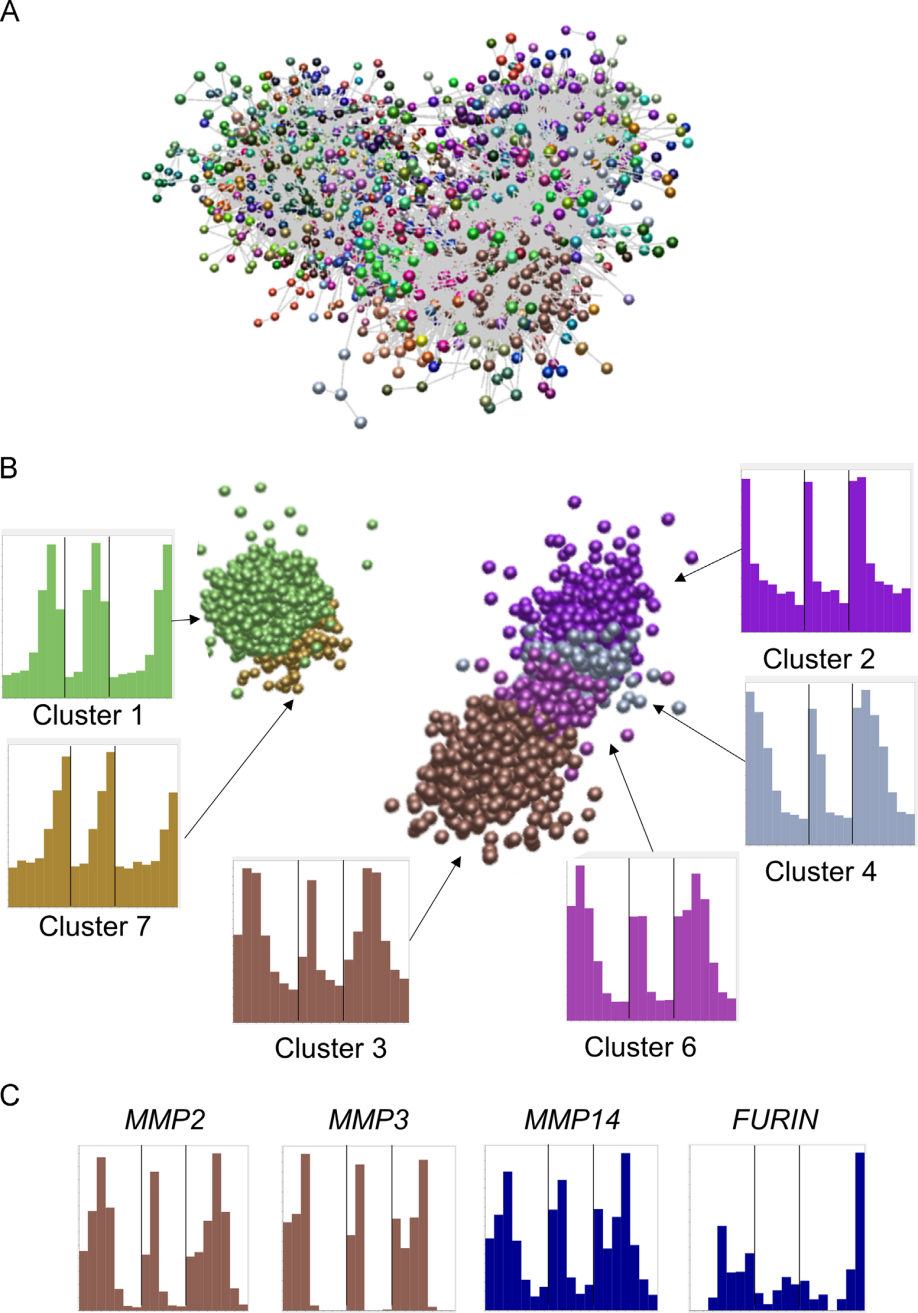
Network visualisation and clustering of gene expression patterns based on transcription initiation during human adipogenesis *in vitro.* Expression levels were derived from transcription start site data [4] available at the FANTOM5 website. Clustering was performed using BioLayout *Express*^3D^ with a correlation coefficient threshold of 0.85 and an MCL inflation value of 2.2. Spheres represent genes and edges correlations in expression patterns between them with a correlation coefficient ≥0.85. (A) Network graph of the main element, showing genes (spheres) and correlations between them of ≥0.85 (grey lines). Nodes in clusters of genes with similar expression patterns as determined by the MCL clustering algorithm, with an inflation value of 2.2, are shown in the same colour. Each replicate was entered separately. (B) Network graph of the main up- and downregulated clusters, with edges removed for ease of visualisation. The average expression pattern of each of the clusters is shown in the histograms. Colours in the histograms are the same as the nodes they represent. Results for individual replicates are shown, separated by lines, and the order of samples across the *X* axis is: Replicate 1: 3 h, 1 day, 2 days, 4 days, 8 days, 12 days, 14 days post induction of differentiation; Replicate 2: 0 h, 2 days, 8 days, 12 days, 14 days post induction; Replicate 3: 0 h, 3 h, 1 day, 2 days, 4 days, 8 days, 12 days post induction. (C) Expression pattern of proteases that cleave fibrillins. *MMP2* (maximum 6000 tpm) and *MMP3* (maximum 160 tpm) were in cluster 3; *MMP14* (maximum 850 tpm) and *FURIN* (maximum 13 tpm) did not cluster.

**Fig. 2:**
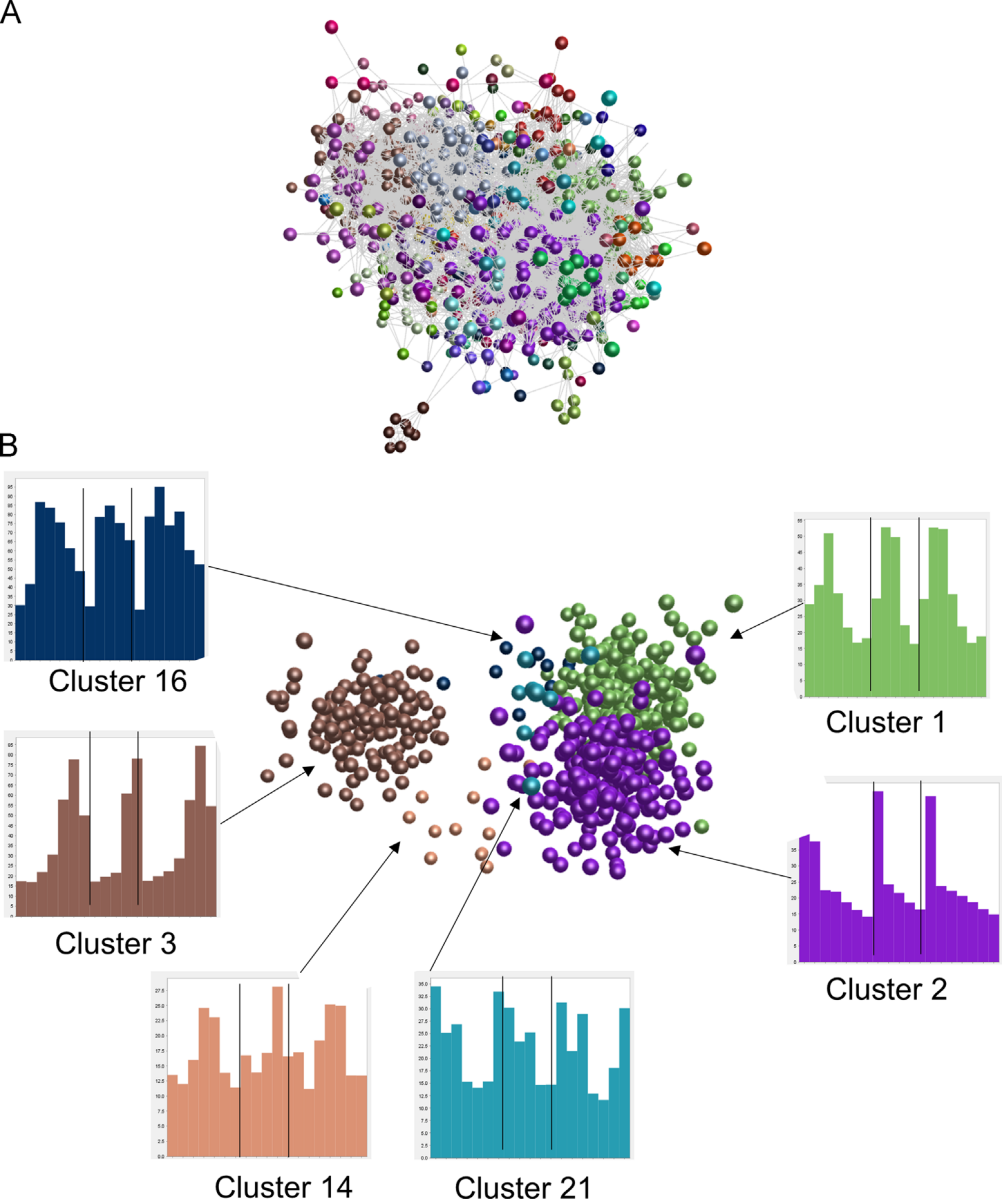
Network visualisation and clustering of gene expression patterns for 1380 transcription factor genes, based on transcription initiation during human adipogenesis *in vitro.* Expression levels were derived from transcription start site data [4] available at the FANTOM5 website. Clustering was performed using BioLayout *Express*^3D^ with a correlation coefficient threshold of 0.62 and an MCL inflation value of 2.2. Spheres represent genes and edges correlations in expression patterns between them with a correlation coefficient ≥0.62. (A) Network graph of the main element, showing genes (spheres) and correlations between them of ≥0.62 (grey lines). Nodes in clusters of genes with similar expression patterns as determined by the MCL clustering algorithm, with an inflation value of 2.2 are shown in the same colour. Each replicate was entered separately. (B) Network graph of the main up- and down-regulated clusters, with edges removed for ease of visualisation. The average expression pattern of each of the clusters is shown in the histograms. Colours in the histograms are the same as the nodes they represent. Results for individual replicates are shown, separated by lines, and the order of samples across the *X* axis is: Replicate 1: 3 h, 1 day, 2 days, 4 days, 8 days, 12 days, 14 days post induction of differentiation; Replicate 2: 0 h, 2 days, 8 days, 12 days, 14 days post induction; Replicate 3: 0 h, 3 h, 1 day, 2 days, 4 days, 8 days, 12 days post induction.

1. Mouse gene expression data were obtained as described in [1]. The list is provided in the Excel workbook, sheet “Mouse genes”, which contains the gene ID, gene symbol, microarray reporter ID and the Pearson correlation coefficient for the correlation of expression pattern across mouse strains with *Fbn1*. A high correlation coefficient indicates that the gene had a very similar expression pattern, that is it was high in epididymal fat of mice where expression of *Fbn1* was also high.

2. Human gene expression during the transition from mesenchymal stem cells to adipocytes in culture was obtained from a publicly available time course of adipogenesis available from the FANTOM5 website, as described in [1]. Gene expression patterns were clustered using BioLayout *Express*^3D^ [1]. Fig. 1A shows the generated network graph of the main element. Fig. 1B shows the up- and downregulated clusters within the same element. The node representing *FBN1* is located in the centre of Cluster 06 (pink). Histograms show expression patterns of these nodes. Fig. 1C shows expression patterns of some key processing enzymes for fibrillin-1. The list of genes in each cluster is provided in the Excel workbook, sheet “Human cluster genes”, which contains the gene sumbol, the cluster number (clusters of four or more genes) and a description of the average expression pattern in the cluster (for clusters with at least 7 nodes; the remaining clusters were too small for meaningful interpretation).
3. A separate file containing gene based transcription factor expression levels was downloaded from the FANTOM5 website for clustering of transcription factor expression patterns. A Pearson correlation coefficient of 0.62 with an MCL inflation value of 2.2 was used to cluster the transcription factors. This ensured that all 1380 available transcription factors were included in the analysis. There were 73 clusters with four or more nodes and 209 genes whose expression pattern did not cluster with at least 3 other genes. Fig. 2A shows the generated network graph of the main element. Fig. 2B shows the up- and downregulated clusters within the same element. Histograms show expression patterns of these nodes. The list is provided in the Excel workbook, sheet “Human TF genes”, which contains the gene symbol, the cluster number and a description of the average expression pattern in the cluster (for clusters with at least 10 nodes).
